# Personality traits prediction based on eye movements while reading manga

**DOI:** 10.3389/fpsyg.2025.1509569

**Published:** 2025-03-31

**Authors:** Yuichi Wada

**Affiliations:** Graduate School of Information Sciences, Tohoku University, Sendai, Japan

**Keywords:** eye tracking, personality, machine learning, gaze behavior, reading, manga

## Abstract

**Background:**

Previous studies utilizing machine learning methods have demonstrated that personal traits can be predicted from eye movement data recorded in real-world situations, such as navigating a university campus or browsing one’s Facebook news feed. The objective of this study was to conceptually replicate and extend these findings in a different type of visual engagement. Specifically, we aimed to predict individuals’ personal traits using eye movements that reflect gaze behaviors while reading manga (Japanese comics).

**Methods:**

We recorded the eye movements of 51 participants as they read manga and trained several machine learning classifiers to predict the levels of each of the self-reported Big Five personality traits from the eye movement features extracted from their reading behavior. The models’ performance was evaluated using cross-validation, and the SHapley Additive exPlanation (SHAP) approach was employed to elucidate the classification model by identifying important features and their impacts on the model output.

**Results:**

Among the Big Five personality traits, only extraversion was predictable. The evaluation results demonstrated that the best model achieved comparable performance with previous literature, with a macro F1 score of 0.49. Analysis of the SHAP value plots showed that a high fixation rate, pupillary response, and blink rate were informative indicators.

**Conclusion:**

The results partially replicated the previously noted associations between eye movement and personality traits. We found that gaze behaviors observed during reading manga are informative of an individual’s extraversion personality trait. We also point out several potential advantages of using manga for gaze-based personality detection.

## 1 Introduction

Eye movements have long attracted considerable research interest as useful indicators of visual perception and human behavior. Some commonly measured eye movements include fixations and saccades. Fixations are defined as eye movements when the eye position remains stationary within a small area of the fovea (typically less than 1°) for some time (less than 50–120 ms). During a fixation, the eyes are focused on a small specific area of the visual field, enabling in-depth processing of visual information. Saccades are rapid eye movements from one fixation point to another, usually lasting 30–80 ms, to reposition the fovea to a new location in the visual field. Practically, the spatial distributions of fixations, fixation duration, saccade amplitude, number of fixations, and scan path pattern are used as typical indices of gaze behavior to “read viewers’ minds” (Zelinsky et al., 2013). Information about where and for how long people look is a rich source of information for inferring what and how we see, think, and act. For example, even when two individuals look at the same visual stimuli, a difference in gaze patterns can be observed, and the causes are derived from differences in the viewers’ attributes, such as gender (Abdi Sargezeh et al., 2019; Mercer Moss et al., 2012; Sammaknejad et al., 2017), age (Kaspar and König, 2012), mental states (Fernández et al., 2013; Jones and Klin, 2013), and cognitive abilities (Hayes and Henderson, 2017).

Among these attributes, this study focused on personal traits. In the last few decades, several studies have found significant associations between the temporal and spatial features of eye movements and various personality traits, including the Big Five personality traits (Agnoli et al., 2015; Perlman et al., 2009; Rauthmann et al., 2012; Wu et al., 2014), behavioral inhibition/activation system (BIS/BAS; Rauthmann et al., 2012), aggressiveness (Bertsch et al., 2017), perceptual curiosity (Baranes et al., 2015; Risko et al., 2012), and trait anxiety (Konovalova et al., 2021). For example, Perlman et al. (2009) showed that individuals who scored high on neuroticism tended to gaze for longer periods at the eye area of faces showing fear compared to those with low levels of neuroticism. Agnoli et al. (2015) found that individuals with higher levels of openness tend to process irrelevant information more positively. This finding suggests that individuals with the openness trait are more inclined to process a broader range of information. This functional connection supports the assertion that open-minded people are receptive to new ideas and driven to expand their experiences by seeking novelty (McCrae and Costa, 1997). More recently, Taib et al. (2020) demonstrated that gaze behavior in response to multiple affective image and video stimuli can predict a wide range of personality traits. Thus, growing evidence shows that gaze preference is associated with various personal traits. However, the relationship between personal traits and gaze is inconsistent (e.g., Harrison et al., 2018).

In many studies investigating the relationship between personality traits and gaze behavior, participants were presented with carefully constructed stimuli for fixed durations, such as static images, sentences, and animations. However, since the properties of gaze behavior recorded in laboratory settings could not sometimes be reproduced in dynamic real-world situations (Foulsham et al., 2011; Tatler, 2014), it remains unclear whether the functional associations of eye movements and personality traits found under controlled laboratory conditions could be generalized to gaze behavior in real-world scenarios.

Hoppe et al. (2018) examined whether a person’s traits can be inferred from eye-tracking data recorded in real-world situations. They recorded the eye movements of 42 participants when shopping on a university campus and employed a machine learning approach to predict the participants’ Big Five personality traits and perceptual curiosity assessed by questionnaires, categorizing them into low, medium, and high score ranges. The Big Five traits comprise the following factors (Costa and McCrae, 1992b): extraversion (e.g., sociable, assertive), agreeableness (friendly, cooperative), conscientiousness (self-disciplined, organized), neuroticism (anxious or depressed), and openness to experience (curious, creative; hereafter referred to as *openness*). They recorded eye movements during browsing and employed a machine learning approach to predict participants’ personality traits, categorizing them into low, medium, and high score ranges. The researchers could predict four of the Big Five personality traits (neuroticism, extraversion, agreeableness, and conscientiousness) and perceptual curiosity from only eye movements. While the levels of prediction accuracy were not very high (up to approximately 15% better than 33% theoretical chance level for the best result), they were significantly above chance level and outperformed several baselines. More recently, Tsigeman et al. (2024) extended the findings of Hoppe et al., where they applied eight machine learning algorithms to the eye movement features recorded while visiting an interactive museum. They predicted four traits out of Big Five with 34–48% precision above 33% chance level.

Woods et al. (2022) investigated whether the Big Five personality traits influence individuals’ browsing tasks on the Web (Facebook news feed). They recorded eye movements during browsing and employed a machine learning approach to predict participants’ personality traits, which were categorized into low, medium, and high score ranges. Woods et al. (2022) reported that the prediction accuracies for conscientiousness and extraversion were better than chance, and the predictive accuracy for extraversion surpassed that of conscientiousness. They also noted that statistical depictions of gaze behavior not associated with the specific content of a Web page were more indicative of extraversion than metrics of gaze behavior calculated based on areas of interest (AOI).

Chen et al. (2023) investigated the possibility of detecting personality traits from users’ eye movements when interacting with a shopping website using a recommendation interface. They built a virtual product comparison website and measured eye gaze behavior when participants chose an item they wished to purchase from multiple product candidates. They developed various machine learning models to predict participants’ personality traits (each classified as high or low) and reported that all traits except extraversion were predicted with an accuracy greater than 0.7. When averaging the performance across all classifiers, conscientiousness yielded the highest predictive accuracy.

The present study attempted to conceptually replicate the findings of the literature cited above, hypothesizing that their findings could be generalized to other types of gazing behaviors. In this study, we define replication success primarily based on whether personality traits can be predicted from eye movement features beyond a baseline level. The aim of the conceptual replication in this study is to verify whether similar results can be obtained under a different stimulus setting compared to previous research. We believe this focus aligns with the goals of conceptual replication, which emphasize the reproduction of broader patterns and relationships (Crandall and Sherman, 2016).

The target situation was reading manga. Manga, Japanese comics, consist of various pieces of information, such as line drawings of characters and text balloons (or speech balloons, representing characters’ discourse), depicted in a manga panel in a monochromatic format. In addition, manga can express the dynamic motion of objects, emotional interactions between characters, and transitions of space and time in stories through successive arrangements of panels (McCloud, 1994). Thus, manga can deliver rich contextual information about the underlying stories based on a mixture of textual and pictorial elements (McCloud, 1994; Natsume, 1997).

Reading manga is a common daily activity for many people, especially younger generations (Clark et al., 2024). Considering this point, we can assume similar outcomes to those of Hoppe et al. (2018) and Woods et al. (2022), in which personal traits produce different gaze patterns when engaging in an everyday task. These gaze patterns can be identified using eye-tracking features while reading manga in combination with machine learning methods. Several studies have investigated gaze behavior during manga reading (Foulsham et al., 2016; Nakazawa, 2002, 2005; Wada, 2023; Wada et al., 2015). However, to our knowledge, no study has directly examined the relationship between readers’ personal traits and gaze behaviors while reading manga using machine learning techniques. This study attempted to answer whether the gaze behavior of individuals while reading manga contains discriminative information and can be used to predict their personality traits.

## 2 Materials and methods

### 2.1 Study participants

As part of their course requirements, graduate and undergraduate students (*N* = 114) participated in a mass screening. Participants were asked to complete an assessment battery measuring various personality characteristics and traits, including an assessment of the Big Five personality traits. Most of these students (*N* = 108) provided permission to contact them for further eye-tracking studies. In the absence of a consensus on how to determine the sample size in machine learning studies, we relied on the sample size of Hoppe et al. (2018) to determine the appropriate sample size for this study. Their sample size was 42 participants in total after excluding eight participants due to data loss because of eye tracker malfunction or too many erroneous samples in their recording of eye-tracking data.

Considering potential technical problems during eye tracking and/or the possibility of participants not showing up, 60 participants were initially recruited for the eye-tracking study. Subsequently, 60 candidates qualified to participate in the eye-tracking study on a first-come-first-served basis. After excluding nine participants, who were found to have at least 20% missing eye-tracking data due to blinks and low data quality, the final sample comprised 51 participants (31 males/20 females, aged 21.7 ± 1.4 years). All participants had normal vision, with some having corrected-to-normal vision with glasses. All experimental procedures were approved by the Institutional Review Board of Tohoku University. Each participant provided written informed consent and received financial compensation.

### 2.2 Apparatus

A Tobii TX-300 eye tracker was used to record gaze data at 250 Hz. The eye tracker was paired with a 23-inch monitor (1920 × 1080 pixels) located approximately 57 cm from the participants. The eye tracker was calibrated to the participants’ eyes using nine fixation points before starting the experiment.

### 2.3 Stimuli

The stimuli used in this study were an original manga story by the author and art by a semi-professional manga writer. It contained 94 panels, 520 Japanese letters in 64 speech balloons, and 13 pages. Digital copies of this manga work were presented on a screen one page at a time, and the resolution of each page was 622 × 880 pixels.

The story depicted a scene in which a female student experienced bullying during her school life. This was announced to candidate participants in advance, and they were informed that they could decline to participate in the experiment if they wished not to read a story with this type of content. None of the participants declined to participate for this reason. Participants were instructed to read the manga story as they usually read manga in everyday life. There was no time limit for the reading process to mimic the natural setting of reading manga. Participants could switch to the next-page display by mouse click at their own pace; however, they could not return to the previous page once they flipped forward to the next one.

### 2.4 Procedure

In this study, participants were instructed to read the manga attentively and naturally, as they would in their usual reading contexts. While we did not include formal manipulation checks (e.g., comprehension test) to assess task engagement or understanding, participants’ eye-tracking data allowed us to identify and exclude participants with unusually sparse or erratic eye movement patterns, which could indicate a lack of engagement.

### 2.5 Questionnaire

The Big Five personality traits were measured using the Japanese version of the Ten-Item Personality Inventory (TIPI-J; Oshio et al., 2012). The TIPI-J serves as a practical tool for efficiently assessing personality traits in a brief manner while maintaining the core structure of the Big Five model. The TIPI-J demonstrates sufficient internal consistency and test–retest reliability, with its construct validity confirmed through correlations with other personality assessments. As mentioned above, the TIPI-J was administered as part of a larger assessment battery. The TIPI-J is beneficial where multiple constructs need to be measured within a limited time frame. For this reason, this scale was used in this study. The items were scored on a 7-point Likert scale, with each Big Five personality trait score ranging from 2 to 14. Personality scores were binned independently into three ranges (low, medium, and high). According to the above bin boundaries, participants were subdivided into low, medium, and high levels for each of the Big Five personality traits. Since the sample size in the eye-tracking study could be too small to be widely distributed in its possible score range or follow a Gaussian distribution, binning was not performed in a data-driven fashion but using predefined middle bin boundaries. The middle bin boundaries were defined as the score percentiles at 1/3 and 2/3, calculated from the data distribution in the mass screening. Therefore, the sample sizes were not perfectly balanced among the three classes. Please see Supplementary Table S1 for details on the data distribution in the current dataset and the bin boundaries between the score ranges. The number of participants in each personality score range are provided in Supplementary Table S2.

### 2.6 Eye-tracking data analysis

The same criteria for erroneous samples (any sample in which the pupil could not be detected or the gaze direction was estimated to be beyond 150% of its range) applied by Hoppe et al. (2018) were used in this study. Nine participants who showed more than 50% erroneous samples in their recordings for more than three pages were excluded from further analysis. For the remaining 51 participants, an average of 88.8 s (SD = 29.1) of eye-tracking data were collected, with minimum and maximum recording times of 48.7 and 197.1 s, respectively.

The eye-tracking features adopted as input features for machine learning were extracted using the Python code publicly available in Hoppe et al. (2018) with a slight modification. First, fixations and saccades were extracted from raw gaze data according to the definitions specified in the above Python code. Fixations were detected based on the dispersion-threshold algorithm (Salvucci and Goldberg, 2000) with a threshold of 2.5% of the tracking range width, and all movements between fixations were defined as saccades. Notably, in our study, the minimum fixation duration was set at 60 ms, whereas it was set at 100 ms in Hoppe et al. (2018). This dispersion threshold was also used to define small and large saccades by comparing the amplitude of a saccade with twice the dispersion threshold.

Hoppe et al. (2018) extracted 207 features related to eye movements from fixations, saccades, blinking information, and pupil diameter. In the present study, the same set of features used by Hoppe et al. (2018) was extracted with a few changes. These changes were as follows: (i) four features (“mean of the mean pupil diameter during saccades,” “var of the mean pupil diameter during saccades,” “mean of the var pupil diameter during saccades,” and “var of the var pupil diameter during saccades”) were not included because of the large amount of missing data, (ii) “mean saccadic peak velocity” was extracted by using slightly different calculation algorithms from the original code, (iii) “mean saccade duration,” which was not extracted in the original code, was newly added. In total, 204 features were extracted and used as input features for the machine learning classifiers in this study. Following Hoppe et al., 204 features were classified into three categories: (1) statistics about fixations, saccades, and blinks, (2) statistics about the spatial pattern of raw gaze data, and (3) information on the temporal course of saccades and fixations (hereafter denoted as the first, second, and third feature groups, respectively). The first feature group consisted of descriptive statistics of fixations, saccades, and blinks (i.e., fixation/saccade rates and the mean/variance/minimum/maximum of the fixation duration). The second feature group represented the spatial distribution of the gaze computed on the x- and y-coordinates of the fixations, with its corresponding pattern of pupil diameter change. The third feature group reflected the global and local patterns of spatiotemporal changes in saccades and fixations. Explanations and details of the feature calculations can be found in the Supplementary material of Hoppe et al. (2018). To improve the performance of the machine learning algorithms, a standard scaler was fitted to the training data, and the training and test samples were standardized to have a mean of 0 and a standard deviation of 1.

Hoppe et al. (2018) extracted features using a sliding window approach with 5–135 s time windows to deal with the unbalanced duration of the recording time of individual data. This approach was not an appropriate way to handle the time series of gaze data in the current study because the total duration (approximately 90 s on average) of the gaze data was too short to produce multiple datasets of a certain time length. Instead, this study utilized a manga page as the unit of gaze data extraction. For each participant and each page, a set of 204 features were extracted. Since 13 pages of manga were presented to each of the 51 participants, our dataset comprised 663 samples. However, the data extracted from the three samples were discarded owing to more than 50% erroneous samples for any feature, resulting in 660 samples for the entire dataset to be analyzed.

### 2.7 Machine learning and model selection

Next, we developed predictive models on a set of 204 eye movement features to reliably predict the participant level of the Big Five personality traits (high, medium, and low) defined by the binning boundaries described above. In this study, three different machine learning models (or classifiers) were employed: random forest (RF), support vector machine (SVM), and ridge regression. We implemented these algorithms using Scikit-learn library in Python (Pedregosa et al., 2011).

RF is an ensemble model of decision trees trained from randomly selected subset features and random sampling of the training set using the bagging method. In each decision tree, a data point falls into a particular leaf depending on its features and is assigned a prediction. The predictions of the data points are then averaged. Hoppe et al. (2018) used RF as a machine learning classifier.

SVM is a supervised learning method that constructs a hyperplane or a set of hyperplanes by employing a nonlinear mapping technique to expand the feature space of the original training data into a higher dimension. Within this augmented dimension, SVM identifies the optimal linear separating hyperplane, which serves as the decision boundary, effectively distinguishing observations belonging to different classes. Woods et al. (2022) reported that SVM can classify the traits of openness, conscientiousness, and extraversion significantly better than chance.

The ridge classifier, a derivative of ordinary least squares linear regression, incorporates L2 regularization to shrink the magnitudes of the regression coefficients, proving advantageous in addressing multicollinearity issues and enhancing the generalization capabilities. Woods et al. (2022) found that it is the best classifier for classifying extraversion.

Although the theoretical chance level should be 0.33 for the current classification, unbalanced numbers of samples in each class could lead to slight deviations in random predictions from the theoretical chance level. In this regard, we compared our classifiers against a baseline model to determine how likely accuracy levels on the current classification predictions were substantial compared with the performance of a dummy classifier with a “stratified strategy” implemented in Scikit-learn, which randomly generates predictions by respecting the training set’s class distribution.

To evaluate the performance of the classifiers, we calculated the macro-averaged F1 score as the evaluation metric. The macro-averaged F1 score is defined as the harmonic mean of precision and sensitivity, in which precision for a certain class indicates how many of the actual class instances were correctly detected, and sensitivity signifies how many of the instances that were predicted to be class instances were correct. The macro-averaged F1 score is computed using the arithmetic mean of all per-class F1 scores, which prevents a classifier from achieving high scores solely by learning that one category occurs more frequently than others, thus offering an advantage over relying solely on accuracy.

To obtain unbiased macro-averaged F1 scores, the classification models were evaluated using a nested (outer: five-fold, inner: five-fold) cross-validation procedure with “StratifiedGroupKfold” from Scikit-learn, selecting hyperparameters on the F1 score metric within the inner loops and collecting F1 scores across the outer loops. Tuned hyperparameters were listed in Supplementary Table S3. The StratifiedGroupKfold is a type of nested cross-validation technique. It combines both stratification and grouping aspects within its folds. A nested cross-validation cycle ensured that data from any participant was present only in either the training or the validation dataset, preventing data leakage and ensuring the model is tested on truly unseen groups of participants during cross-validation. Stratification ensures that five different data splits (folds) were chosen to contain an approximately equal number of samples for each class, crucial for balanced model evaluation. From the aforementioned cross-validation procedure, a total of five F1 scores were obtained in the outer loop. The average of these scores was considered the performance measure for a single analysis. To strengthen the stability of the classification performance, we ran the cross-validation procedure 20 times with different splits of data into training and testing sets, and the final F1 score was defined as the average estimate over all 20 runs.

## 3 Results

### 3.1 Model performance

Table 1 summarizes the performance achieved across the classifiers for each personality trait. One can observe that three of the four classifiers classified the participants’ extraversion trait well above chance. The ridge classifier yielded the best F1 score of 0.490, and the SVM yielded an F1 score of 0.466. The RF classifier had an F1 score of 0.405, which was the lowest among all the classifiers tested.

**Table 1:** Mean (SD) F1 scores of 20 instances of the three classifiers and a baseline model per each personality trait.

Trait	Baseline	RF	SVM	Ridge
Extraversion	0.330 (0.018)	0.405 (0.025)	0.466 (0.025)	0.490 (0.026)
Conscientiousness	0.333 (0.025)	0.281 (0.023)	0.318 (0.025)	0.296 (0.022)
Agreeableness	0.333 (0.011)	0.292 (0.025)	0.312 (0.015)	0.331 (0.016)
Neuroticism	0.335 (0.016)	0.292 (0.027)	0.320 (0.022)	0.317 (0.015)
Openness	0.332 (0.015)	0.321 (0.031)	0.331 (0.027)	0.326 (0.020)

RF, random forest; SVM, support vector machine; Ridge, Ridge classification.

To assess the performance of the classifier, we performed a series of permutation tests (applying 1,000 permutations) on the 20 data points obtained for each classifier through the procedure described above. Here we adjusted the resulting *p*-values for multiple comparisons using the Bonferroni correction method. Corrected alpha values below 0.05 were deemed statistically significant. For the classification of extraversion, all classifiers exhibited a statistically significant improvement in the F1 score compared with the baseline. The ridge classifier showed the highest prediction accuracy, but there was no significant difference compared to the performance of the SVM. These two classifiers demonstrated significantly higher classification performance than the random forest classifier.

For the remaining four traits, no classifier predicted personality score ranges significantly above the performances of the random baseline model, and for three of the four traits (conscientiousness, agreeableness, and neuroticism), some of the classifiers performed even worse than the baseline. Therefore, the main interest of the results reported below lies in investigating in more detail how eye movement parameters are linked to the individual score ranges of extraversion.

Woods et al. (2022) noted that non-AOI-based gaze metrics, which were not associated with the specific content of Web pages, were indicative of extraversion, whereas AOI-based information derived from gaze behavior toward a particular category or area of stimuli was not informative of extraversion. To examine whether the same situation occurs in the present study, we built and evaluated our classifiers separately with three groups of feature categories: (1) statistics about fixations, saccades, and blinks; (2) statistics about the spatial pattern of raw gaze data; and (3) information on the temporal course of saccades and fixations. Among these three feature groups, the second group can be regarded as features that are extracted in a manner similar to the AOI-based information because the characteristics of the features included in this group are dependent on the arrangement and types of objects present within the observer’s field of view during gaze measurement. The performance in predicting extraversion achieved across the three feature groups for each classifier is summarized in Table 2. We conducted a series of permutation tests for each group of feature categories. For the first and second feature groups, the results revealed that all three classifiers significantly predicted the level of extraversion compared with the baseline. For the third feature group, no classifiers were successful in predicting the degree of extraversion. Next, we conducted a series of permutation tests for each of the three classifiers, excluding the baseline model to determine whether there was a difference in prediction performance between the first feature group and the second feature group. Results showed that the F1 scores using the first feature group were significantly higher than those using the second feature group for all the classifiers tested.

**Table 2:** Mean (SD) F1 scores of 20 instances of the three classifiers and a baseline model evaluation by each feature group.

Feature group	Baseline	RF	SVM	Ridge
1st feature group	0.336 (0.016)	0.424 (0.031)	0.462 (0.021)	0.486 (0.024)
2nd feature group	0.329 (0.019)	0.388 (0.027)	0.374 (0.024)	0.390 (0.018)
3rd feature group	0.331 (0.020)	0.319 (0.012)	0.324 (0.010)	0.331 (0.014)

To determine whether similar F1 scores were observed across trait levels (low, medium, and high), Woods et al. (2022) reported the F1 scores for each trait level. Following this, we further assessed the predictive performance for each classifier using the first feature group. As shown in Table 3, all classifiers produced similar patterns of F1 scores across trait levels, resulting in incremental improvements across the low, medium, and high categories. The standard deviation of the F1 scores across the 20 runs indicated the degree of variability in performance across iterations with different training/test datasets. Considering that the standard deviation values were similar across the classifiers and trait levels, we can ascertain that the classifiers’ performance was relatively superior when predicting a high level of extraversion. If the model accurately predicts personality traits, it is expected that misclassifications would predominantly occur between adjacent categories (e.g., high-medium or medium-low) rather than extreme ones (e.g., high-low). To examine this point, the confusion matrices of the four classifiers comparing the true and predicted extraversion trait levels are provided in the Supplementary Figure S1. From the results of the confusion matrices for ridge regression and SVM, although a slight tendency to skew toward the high level is observed, there is no significant difference in prediction performance among the three categories. Additionally, it can be inferred that misclassifications primarily occur between adjacent categories. This suggests that these models were able to predict the levels of extraversion in a balanced manner.

**Table 3:** Mean (SD) F1 scores of 20 instances of three classifiers and a baseline model evaluation by each trait level.

Trait level	Baseline	RF	SVM	Ridge
Low	0.325 (0.042)	0.357 (0.067)	0.449 (0.093)	0.454 (0.087)
Medium	0.347 (0.040)	0.422 (0.068)	0.454 (0.066)	0.491 (0.083)
High	0.311 (0.046)	0.432 (0.052)	0.504 (0.072)	0.515 (0.082)

### 3.2 SHAP values of features

To facilitate the interpretation of the classification model, the SHapley Additive exPlanations (SHAP) algorithm (Lundberg et al., 2018) was applied to the SVM classifier using the first feature group to gauge the influence of each eye-movement parameter on the model predictions. The SHAP algorithm calculates SHAP values for each feature, representing the relative importance of the features in the predictive model. We did not focus on the ridge classifier, which exhibited the best performance, because of the lack of official support for the “predict_proba” function for the ridge classifier when implemented in Scikit-learn, which is necessary for computing SHAP values. Here, the absolute SHAP values of the 204 features were averaged across the participants in the validation dataset. Since prediction results depended on the random seed used for the separation of training and test data, a different set of SHAP values was calculated for each run of the cross-validation procedure, and they were aggregated across all 20 runs.

**Figure 1:**
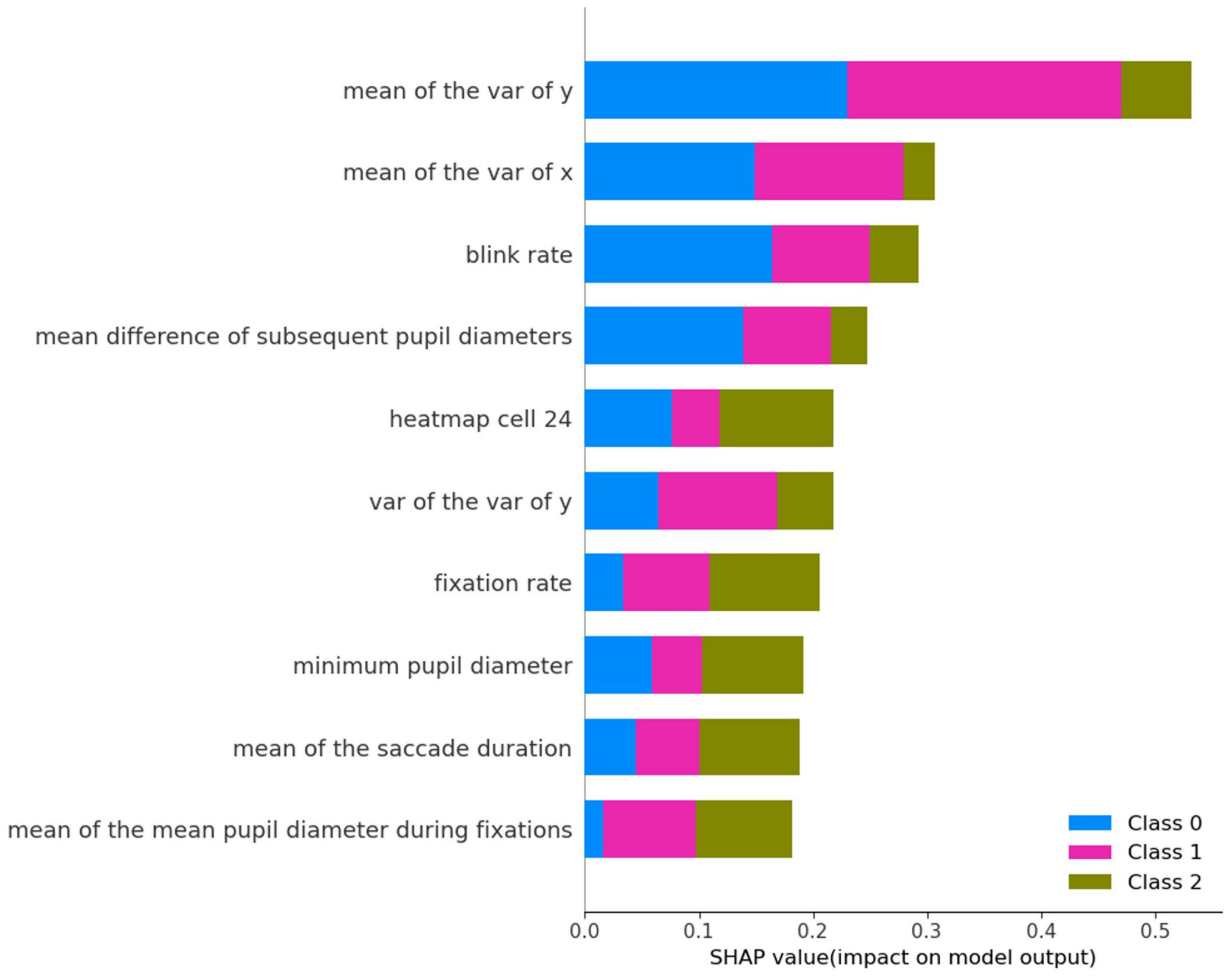
SHAP global feature importance plot: the top 10 features of the SVM model. Bar plot of mean absolute SHAP values of individual features.

**Figure 2:**
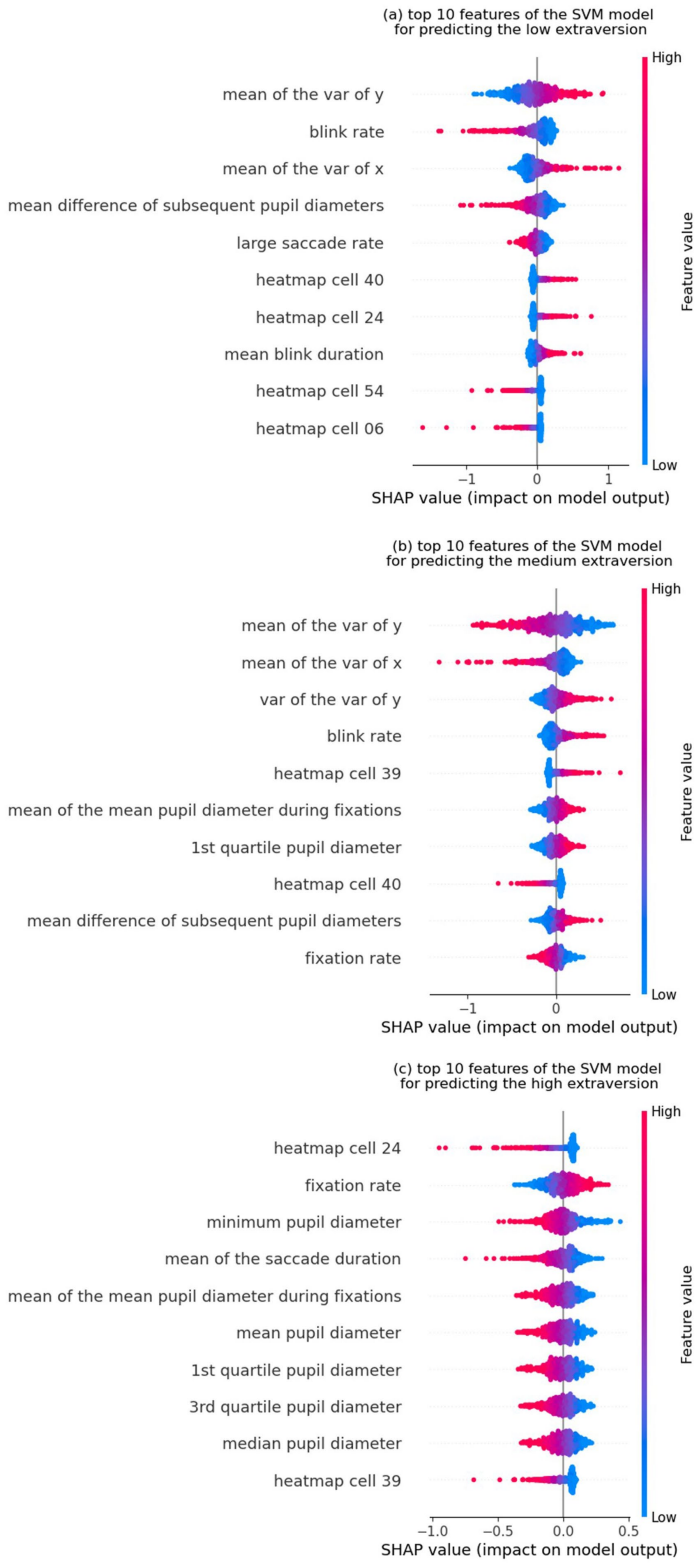
SHAP local explanation summary plot for the top 10 features of the SVM model for predicting **(a)** the low extraversion (or high introversion), **(b)** medium extraversion, and **(c)** high extraversion (or low introversion) levels. The color change in the summary plot (from left to right) of each feature from blue to red indicates a positive influence on classification into the targeted class.

Figure 1 shows the top 10 features used to predict extraversion, sorted in ascending order by their SHAP values (where greater values reflect greater influence in model predictions). Among these features, “mean of the variance of y,” “mean of the variance of x,” and “blink rate” were the top three influential features. Figures 2a–c represent the SHAP summary plots and the local explanation exhibiting the direction of the relationship between each feature and the low, medium, and high levels of extraversion, respectively. The SHAP local summary plots are represented as violin plots aligned with the corresponding SHAP values along the x-axis. For each of the three levels (low, medium, and high) of the extraversion personality trait, the top 10 features are displayed in descending order, with the most impactful features at the top. Notably, the variety of features differed among the three levels.

Figure 2a shows that the “mean of the variance of y,” “blink rate,” and “mean of the variance of x” provide information for predicting the low extraversion (or high introversion) level. The widespread distribution of fixations on the manga page seems to represent the characteristic gaze patterns of introverted participants. Additionally, a smaller change in pupil diameter contributes to the prediction of low extraversion. In Figure 2b, two of the top three informative features (“mean of the variance of x” and “mean of the variance of y”) are the same as the ones found in Figure 2a, indicating that these features have a substantial impact on the prediction of the medium extraversion level. However, the direction of the effects was contrary to the prediction of the low extraversion level. The small variance in fixation locations was closely associated with a medium extraversion level. Figure 2c illustrates the nonuniformity of fixation positions in the horizontal direction (“heatmap cell 24”); high fixation rates and smaller pupil sizes are associated with high extraversion levels. To further validate the effectiveness of the analysis, it would be beneficial to compare SHAP values with the feature importance scores derived from the Random Forest (RF) model. SHAP global feature importance plot for the top 10 features of the Random Forest (RF) model (Supplementary Figure S2) and SHAP local explanation summary plot (Supplementary Figure S3) are provided in the Supplementary material. From these results, although there are differences in ranking, it can be observed that several of the top 10 most influential features overlap between the two models. A high degree of consistency in SHAP values between the two models strengthens the reliability and interpretability of the model’s feature selection process.

In addition to the commonly reported F1 scores used to evaluate machine learning performance, we also present correlation coefficients between personality scores and various eye movement features. In this analysis, the total score for each trait, calculated as the sum of two item scores ranging from 2 to 14, was correlated with eye movement metrics recorded across 13 pages of manga reading. For each personality dimension, we extracted the top 10 features with the highest absolute correlation coefficients and compiled them into a list, which is presented in Supplementary Table S4. According to this table, each personality dimension exhibited correlations with at least one eye movement metric, with correlation coefficients ranging from 0.2 to 0.3, distributed within a similar range across all personality dimensions. Moreover, the features showing correlations with eye movement metrics were relatively distinct across different personality dimensions. The results of the correlation analysis indicate that eye movements are associated with four of the five personality dimensions, excluding extraversion. At the same time, these findings suggest that, for the four personality dimensions other than extraversion, the eye movement metrics that showed significant correlations with personality scores do not necessarily carry sufficient predictive value for personality levels in a machine learning approach. We also evaluated possible gender differences for the parameters of eye movements. We conducted independent t-tests to examine whether there were significant differences in eye movement parameters between male and female participants, but none were found even prior to adjusting significance levels with the Bonferroni correction.

## 4 Discussion

This study attempted to conceptually replicate previous findings demonstrating that the gaze behavior of individuals contains discriminative information that can be used to predict their personality traits. We presented evidence that an individual’s eye movement pattern extracted from reading manga is informative of one of the Big Five personality traits: extraversion.

Among the Big Five personality traits, only extraversion was predictable. The result that extraversion was significantly predictable is consistent with the findings of Hoppe et al. (2018), Tsigeman et al. (2024), and Woods et al. (2022). The methodological differences between the present and previous studies are substantial. Nevertheless, the results of the present study demonstrate the feasibility of predicting the levels of participants’ extraversion with a relatively moderate accuracy while participants are simply reading manga. In fact, the F1 score for predicting the level of extraversion (0.490) is comparable to that in prior works (0.486 in Hoppe et al., 2018; 0.457 in Tsigeman et al., 2024; 0.476 in Woods et al., 2022) where eye-tracking measures extracted from everyday tasks were used as input features to machine learning models.

For the question of whether the specific eye movement features used to predict extraversion also correspond with those identified in previous research, several common informative features were found in this study and previous literature. Rauthmann et al. (2012) showed that viewers’ extraversion was related to a shorter dwelling time and a higher number of fixations while viewing computer-generated abstract stimuli. The latter result is consistent with the interpretation of the SHAP values in this study, showing that a high fixation rate is linked to a high level of extraversion. This personality trait has been associated with cognitive processes linked to external orientation and the proactive pursuit of novel information (Aluja et al., 2003; Costa and McCrae, 1992b). Such a behavioral pattern may result in an ample distribution of fixations in individuals with high levels of extraversion. The SHAP analysis in the current study also revealed that features related to the pupillary response and blink rate were informative indicators, which is partly in accordance with the results of Taib et al. (2020) and Hoppe et al. (2018).

Woods et al. (2022) noted that statistical depictions of gaze behavior that are not associated with the specific content of Web pages were indicative of extraversion, whereas metrics of gaze behavior derived from specific metrics calculated based on AOI were not. However, the findings of our study showed that the gaze features calculated from AOI-based information (the second feature group mentioned above) could predict extraversion significantly; however, the contribution of the gaze features extracted from the statistical depictions of gaze behavior (the first feature group) was much larger. The predictive effects for extraversion based on the statistical depictions of gaze behavior were substantial, which aligns with the findings of Woods et al. (2022). By contrast, in the present study, the predictive effects of AOI-based information were weaker but still substantial compared with those of statistical depictions of gaze behavior for predicting extraversion. The informative features observed in this study also differ from those identified by Hoppe et al. (2018). For example, saccade-based *n*-grams (Bulling et al., 2011) and heatmap features (Risko et al., 2012), which were the most important feature classes in Hoppe et al. (2018), were not included in the important features listed in the current study. These discrepancies may have been, at least partly, due to differences in the experimental settings and/or the nature of the stimuli viewed by participants. In this study, eye movements were monitored during manga reading, facilitating direct comparability, as the manga content presented to the participants was uniform. Meanwhile, in Woods et al. (2022), AOIs were defined as corresponding to several areas on a Facebook news feed screen. Hence, although the spatial layouts of AOIs in Woods et al. (2022) were almost the same, the information contents of AOIs (texts and images) were different for items (color, spatial frequencies, subject matter, and so on) across each participant. Similarly, in Hoppe et al. (2018), the eye movements of participants were tracked while viewing different sights while walking around campus for shopping. These varieties of view content might introduce a significant degree of variability in visual behavior that is not directly associated with the participant’s personality and thus cause difficulty in the prediction of personality traits. Therefore, caution should be exercised when comparing the findings of different studies.

In terms of predictive accuracy, the superiority of extraversion among the Big Five personality traits is consistent with the results of previous studies. Hoppe et al. (2018) found that the prediction of extraversion was more accurate than the other three reliably predicted traits (conscientiousness, agreeableness, and neuroticism). Similarly, Tsigeman et al. (2024) noted that the most predictable trait in their experiment was extraversion. Woods et al. (2022) reported that the prediction performance for conscientiousness and extraversion was better than chance and that the predictive accuracy for extraversion surpassed that of conscientiousness. With regard to the difficulty of predicting conscientiousness, Woods et al. (2022) speculated that this could be because their classifier performed markedly worse when predicting low levels of conscientiousness. According to their explanation, individuals exhibiting a lower level of conscientiousness would likely demonstrate diminished response consistency owing to a reduced inclination toward task fidelity, potentially leading to greater score variability. Consequently, it is conceivable that the predictive accuracy for the low conscientiousness category would be low compared with the other two categories. However, such a tendency was not observed in our results. Rather, it can be discerned that the dispersion of scores in the high conscientiousness category was larger compared with the other two categories based on the fact that the standard deviation of scores in the high conscientiousness category was approximately 1.7 times as high as that for the low conscientiousness category. Hence, score variability does not appear to be the cause of our failure to establish a strong predictive model for participants’ conscientiousness levels.

Regarding the other three traits (neuroticism, agreeableness, and openness), Hoppe et al. (2018) showed that their classifier performed well above chance for neuroticism and agreeableness but not for openness. Tsigeman et al. (2024) also found that these three traits were reliably predicted from eye movements. Meanwhile, the classifiers used by Woods et al. (2022) failed to predict these three traits better than chance. Hence, we can say that openness was the only trait that could not be significantly classified in these two related studies. However, according to Rauthmann et al. (2012), individuals characterized by high levels of openness tend to exhibit longer fixation and dwelling times during the observation of abstract animations. Furthermore, Taib et al. (2020) demonstrated that all Big Five personality traits could be accurately predicted using eye movement features in response to multiple affective image and video stimuli. Thus, for the four personality traits, excluding extraversion, there are mixed results on whether eye movement data can be used to make accurate and statistically significant classification, and no consensus has been reached. Various factors, such as the types of experimental stimulus settings presented to participants, the presence or absence of task demands, the duration of eye-tracking data collection, and the types of eye movement features utilized in machine learning algorithms, vary among previous studies, making comparisons of results challenging.

Nonetheless, we can claim that among the Big Five personality traits, extraversion readily reflects individual differences in the statistical metrics of eye movements, which are extracted from a variety of visual sources. Crucially, the underlying cause of our classifiers’ subpar performance in predicting the other four personality traits remains elusive at this point, as we cannot determine whether it stems from the limitations inherent in the experimental design, specifically the task of manga reading, or from the potential absence of a correlation between these traits and the eye movement patterns observed during manga reading.

The eye-tracking data used in this study were tracked for an average of approximately 90 s. While not as brief as the 20-s measurement period employed by Woods et al. (2022), this interval marks a considerable shortening of the time scales of approximately 10 min utilized in similar studies (Hoppe et al., 2018; Taib et al., 2020). This study, which demonstrates the ability to predict certain personality traits from relatively short data measurements of approximately 90 s, provides valuable insights into the practical application of eye-tracking data, as mentioned above. Additionally, the act of reading manga is accessible to a wide age range, from children to adults, and is generally perceived as a leisure activity by many people. This suggests that the mental burden associated with this task was low. These factors make the manga reading task advantageous compared with the experimental settings used in previous research.

The current study has certain limitations. First, the data were obtained from a single manga work; therefore, their generalizability to other manga works is limited. Further studies with different contents of manga works as well as similar types of reading materials, such as comic graphic novels and bandes dessinées, can enhance the generalizability of our findings. Second, we did not adopt the AOI-based approach, which categorizes and analyzes eye gaze data directed toward specific visual areas or objects. Not introducing this analytical method allows for the analysis of eye-tracking data in a form that does not depend on specific stimuli, thereby enhancing methodological flexibility. In contrast to the present study, Woods et al. (2022) reported that several AOI-based features affect the prediction of conscientiousness. Gao et al. (2023) also showed that participants’ eye movement behavior toward virtual objects in a virtual reality classroom setting provides discriminative information for predicting their gender. These findings suggest that how participants allocate their visual attention to different objects reflects distinct psychological attributes. By incorporating AOI-based features into machine learning models’ predictions, future research may enhance the predictive accuracy for personality traits other than extraversion, which did not reach statistically significant levels in the current analysis. Additionally, since manga is presented as static images, setting AOIs is relatively easier compared with data measurement scenarios involving the dynamic motion of elements, such as Web browsing, video viewing, or everyday activities. Furthermore, eye movement data were collected from university students who were presumed to have extensive experience with reading manga. However, this study did not investigate potential differences in eye movement behavior based on demographic characteristics (e.g., gender and age) or the level of reading experience, which might have influenced the classifiers’ ability to predict personality traits. Future research should consider these factors to enhance our understanding of the utility and potential of predicting personality traits through eye movements.

Finally, several methodological limitations should be noted, one of which is the reliance on short-form measures for assessing the Big Five personality traits. Although the short-form measures provide a time-efficient assessment, its brevity comes at the cost of reduced granularity (Donnellan et al., 2006). With only a limited number of items per domain, it may fail to fully represent the complex and multidimensional nature of the Big Five personality traits. Future studies would benefit from employing a more comprehensive, multi-faceted measure (e.g., the NEO-PI-R; Costa and McCrae, 1992a) to assess personality traits and investigating the relationships between these facets and gaze patterns, which may lead to significantly improved predictive accuracy. In this study, we intentionally designed the task situation to closely resemble real-life conditions; therefore, we did not conduct a manipulation check to assess the extent to which participants were engaged in the task. However, we acknowledge that individual differences in engagement attitudes, potentially influenced by personality traits, might still have impacted the results. In relation to this point, among the Big Five personality dimensions, conscientiousness has been suggested to potentially reflect the degree of participants’ engagement in experimental tasks (Woods et al., 2022). As a future research direction, it would be worthwhile to consider obtaining additional measures, such as indices assessing participants’ comprehension of the manga’s storyline and their level of interest in manga reading, in conjunction with eye movement data.

## 5 Conclusion

This study aimed to conduct a conceptual replication of previous research (Hoppe et al., 2018; Tsigeman et al., 2024; Woods et al., 2022) by focusing on the gaze behavior of individuals while reading manga as a different type of eye gaze behavior from that studied in previous research. The present results partially replicated the previous findings. The gaze data extracted from manga reading contained discriminative information for predicting extraversion. Conceptual replications provide information about the generalizability of inferences across different populations and ways of operationally defining constructs (Crandall and Sherman, 2016). Such replication is timely as empirical research on the association between personality and oculomotor behavior has been limited to date. In addition, the issue of reproducibility in scientific research has been a topic of discussion for the past few decades, particularly in the field of psychology. This topic is often discussed in relation to the misuse of statistical tests; however, scientific findings based on machine learning methods are by no means immune to reproducibility concerns. Hence, we examined gaze patterns while reading manga using machine learning algorithms and were able to replicate at least part of the results of the previous literature. From this perspective, the findings of this study make a significant contribution to the relevant literature.

## Data Availability

The raw data supporting the conclusions of this article will be made available by the authors, without undue reservation.
