# Supplementary material for: Personality traits prediction based on eye movements while reading manga
Source: Front Psychol. 2025 Mar 31;16:1509569. doi: 10.3389/fpsyg.2025.1509569 (PMC11994591; doi:10.3389/fpsyg.2025.1509569)

Supplementary Material

# Supplementary Figures and Tables

## Supplementary Table S1

Personality score ranges for each personality trait. The participants' raw scores were categorized into distinct score ranges based on the following criteria. Participants whose raw scores equaled or fell below boundary 1 were allocated to score range low, while those exceeding boundary 1 but equaled or fell below boundary 2 were placed into score range middle. Participants surpassing boundary 2 were categorized into score range high.

| trait | Mean (SD) | | |  |  |
| --- | --- | --- | --- | --- | --- |
|  | Low | Medium | High | boundary 1 | boundary 2 |
| Extraversion | 4.95 (1.07) | 7.71 (0.73) | 11.47 (1.31) | 6 | 9 |
| Conscientiousness | 4.02 (0.92) | 6.41 (0.50) | 9.66 (1.56) | 5 | 7 |
| Agreeableness | 7.52 (1.55) | 10.45 (0.50) | 12.44 (0.64) | 9 | 11 |
| Neuroticism | 5.52 (1.36) | 8.43 (0.50) | 11.28 (1.29) | 7 | 9 |
| Openness | 5.70 (1.18) | 8.25 (0.44) | 10.92 (1.09) | 7 | 9 |

## Supplementary Table S2

The number of participants in each personality score range.

|  | Extraversion | Conscientiousness | Agreeableness | Neuroticism | Openness |
| --- | --- | --- | --- | --- | --- |
| Low | 13 | 20 | 20 | 24 | 19 |
| Medium | 18 | 14 | 18 | 16 | 13 |
| High | 20 | 17 | 13 | 11 | 19 |

## Supplementary Table S3

Tuned hyperparameters for each classifier.

| Classifier | Parameter | Values |
| --- | --- | --- |
| random forest (RF) | n_estimators | [10, 30, 50, 100, 300] |
|  | max_features | ['sqrt', 'log2'] |
|  | max_depth | [10, 20, 30, 40, 50] |
|  | criterion | ['gini', 'entropy'] |
| support vector machine (SVM) | C | [0.01, 0.1, 1, 10, 100] |
|  | kernel | ['linear', 'rbf', 'poly', 'sigmoid' ] |
|  | gamma | [0.001, 0.1, 1] (not available for 'lienar' kernel) |
|  | degree | [2, 3, 4] ('poly' kernel only) |
| ridge regression | alpha | [500, 300, 100 ,10, 1, 0.1, 0.01, 0.001] |

## Supplementary Table S4

Correlation coefficients between personality scores for each trait and the average eye movement metrics measured across 13 pages of manga reading. The table includes the smallest feature set containing the 10 features with the highest correlations for each trait, highlighted in bold. N-gram features are labeled sacc. movements if based only on saccades and SF. movements if both of fixations and saccades are considered.

| **feature** | **Extra** | **Agree** | **Cons** | **Neuro** | **Open** |
| --- | --- | --- | --- | --- | --- |
| 1st quartile pupil diameter | -0.219 | 0.150 | 0.010 | 0.062 | **-0.302** |
| heatmap cell 00 | -0.193 | **0.295** | 0.229 | -0.153 | -0.108 |
| heatmap cell 02 | -0.019 | **-0.252** | -0.024 | 0.116 | 0.115 |
| heatmap cell 04 | 0.233 | -0.037 | **0.332** | -0.262 | 0.127 |
| heatmap cell 11 | **0.298** | -0.076 | -0.077 | 0.159 | 0.169 |
| heatmap cell 18 | 0.100 | **-0.264** | -0.239 | 0.154 | -0.099 |
| heatmap cell 20 | **0.378** | -0.069 | -0.161 | -0.057 | 0.181 |
| heatmap cell 21 | 0.224 | -0.039 | 0.154 | **-0.280** | -0.016 |
| heatmap cell 22 | 0.172 | -0.146 | 0.058 | **-0.292** | -0.009 |
| heatmap cell 24 | **-0.276** | 0.082 | 0.118 | 0.098 | 0.003 |
| heatmap cell 26 | -0.061 | 0.116 | -0.018 | -0.027 | **0.383** |
| heatmap cell 31 | 0.133 | **-0.269** | -0.117 | **0.288** | 0.033 |
| heatmap cell 35 | **-0.300** | 0.132 | 0.082 | 0.005 | -0.265 |
| heatmap cell 37 | 0.053 | **0.340** | -0.222 | 0.091 | 0.194 |
| heatmap cell 40 | **-0.302** | 0.197 | -0.170 | 0.085 | -0.190 |
| heatmap cell 44 | -0.038 | **-0.263** | **-0.460** | **0.338** | -0.222 |
| heatmap cell 49 | -0.083 | 0.074 | **-0.279** | 0.230 | **-0.332** |
| heatmap cell 55 | -0.066 | **0.247** | -0.191 | 0.062 | -0.097 |
| heatmap cell 59 | -0.039 | 0.139 | **0.327** | 0.012 | 0.272 |
| heatmap cell 60 | 0.257 | **-0.309** | 0.097 | -0.070 | 0.185 |
| least frequent 1-gram SF movement | 0.213 | -0.118 | -0.129 | 0.216 | **0.311** |
| least frequent 1-gram sacc. movement | 0.190 | -0.148 | **-0.276** | -0.046 | -0.073 |
| least frequent 2-gram sacc. movement | -0.081 | **-0.332** | -0.203 | 0.091 | -0.066 |
| max frequency 4-gram SF movements | 0.177 | -0.003 | **-0.291** | 0.070 | 0.001 |
| mean of the mean of subsequent angles | 0.121 | -0.097 | 0.039 | **-0.281** | -0.079 |
| mean of the mean pupil diameter during fixations | -0.217 | 0.148 | 0.014 | 0.056 | **-0.292** |
| mean of the saccade duration | **-0.270** | 0.008 | 0.007 | 0.215 | 0.125 |
| mean of the var of x | **-0.272** | 0.029 | -0.085 | 0.185 | -0.008 |
| mean of the var of y | -0.196 | -0.042 | 0.088 | **0.379** | 0.135 |
| mean pupil diameter | -0.220 | 0.146 | 0.005 | 0.061 | **-0.297** |
| median pupil diameter | -0.217 | 0.145 | 0.001 | 0.059 | **-0.298** |
| min blink duration | **-0.293** | 0.157 | 0.018 | -0.041 | -0.217 |
| min frequency 1-gram SF movements | -0.007 | 0.100 | **-0.297** | -0.149 | -0.023 |
| minimum pupil diameter | **-0.339** | 0.218 | -0.015 | 0.070 | **-0.329** |
| most frequent 1-gram SF movement | 0.004 | -0.129 | -0.066 | **0.290** | 0.183 |
| most frequent 1-gram sacc. movement | -0.001 | -0.095 | -0.072 | **0.335** | -0.052 |
| range of frequencies of 4-gram SF movements | 0.181 | -0.004 | **-0.305** | 0.057 | 0.011 |
| ratio of large saccades | 0.158 | 0.072 | -0.173 | **-0.284** | -0.260 |
| ratio of small saccades | -0.158 | -0.072 | 0.173 | **0.284** | 0.260 |
| small saccade rate | -0.017 | -0.113 | **0.290** | 0.120 | **0.312** |
| std x | 0.087 | -0.053 | **0.279** | -0.114 | **0.358** |
| var blink duration | -0.136 | **-0.235** | -0.107 | 0.099 | 0.046 |
| var of the var of x | **-0.284** | 0.078 | -0.031 | 0.015 | -0.064 |

## Supplementary Figure S1.

## Confusion matrix in predicting extraversion for the four classifiers.


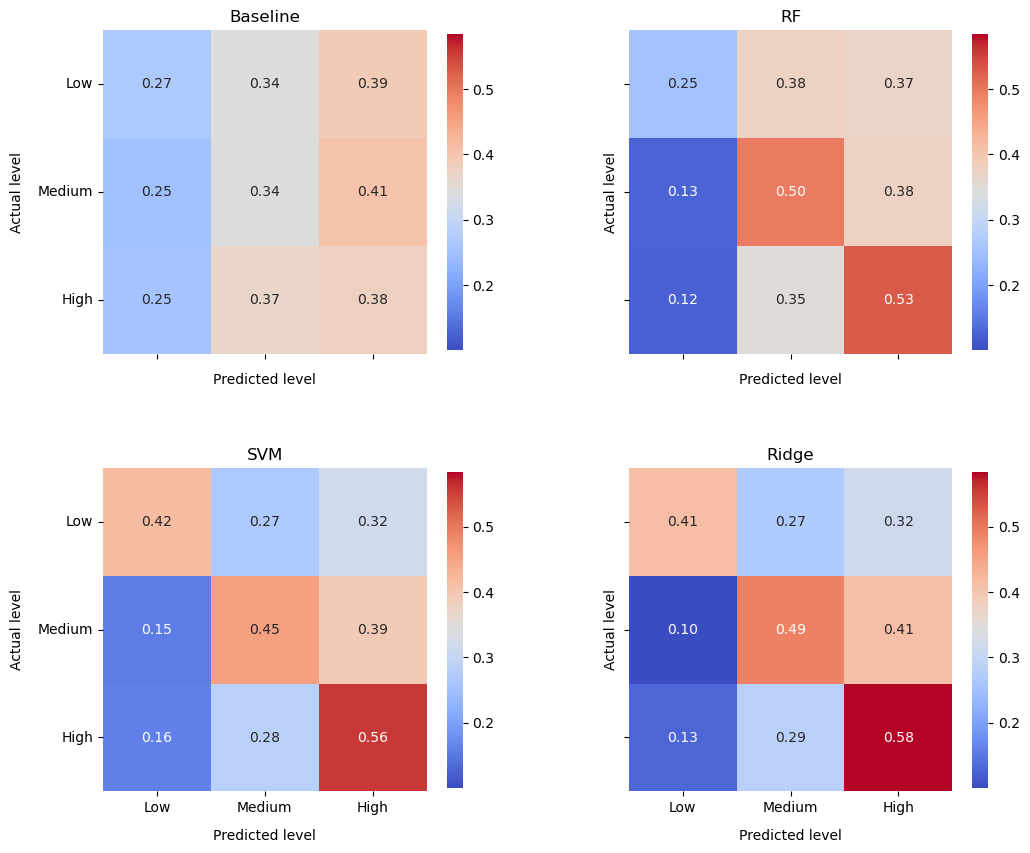


RF, random forest; SVM, support vector machine.

## Supplementary Figure S2.

## SHAP global feature importance plot: the top 10 features of the RF model. Bar plot of mean absolute SHAP values of individual features.


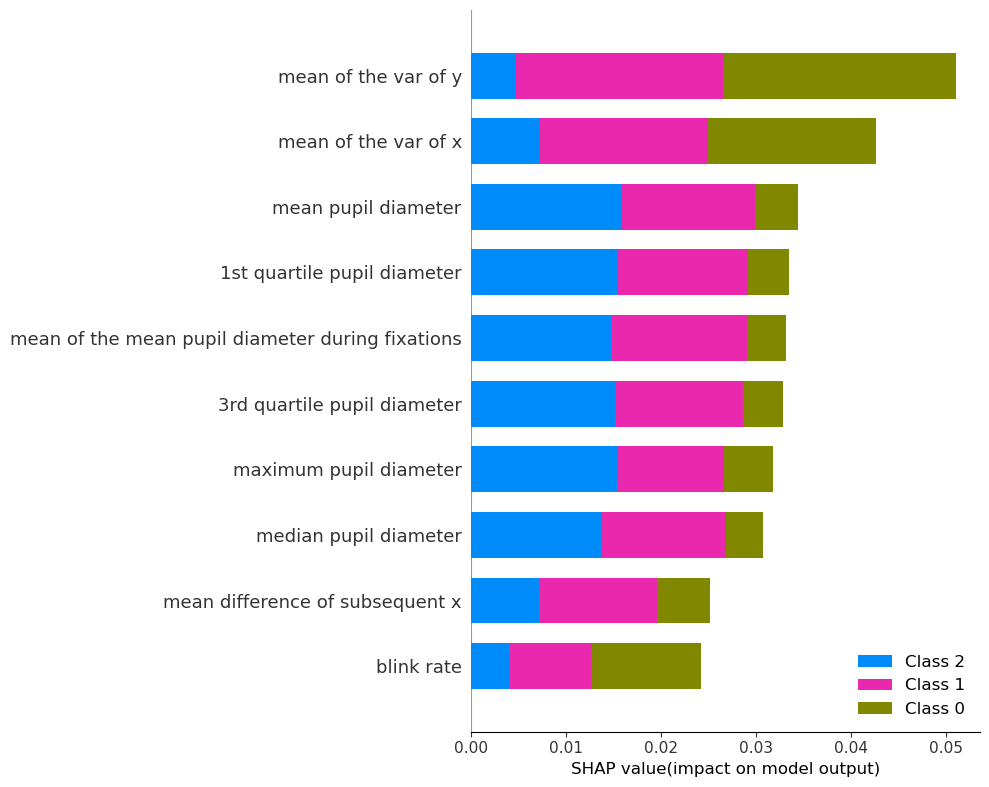


## Supplementary Figure S3.

SHAP local explanation summary plot for the top 10 features of the RF model for predicting (A) the low extraversion (or high introversion), (B) medium extraversion, and (C) high extraversion (or low introversion) levels. The color change in the summary plot (from left to right) of each feature from blue to red indicates a positive influence on classification into the targeted class.


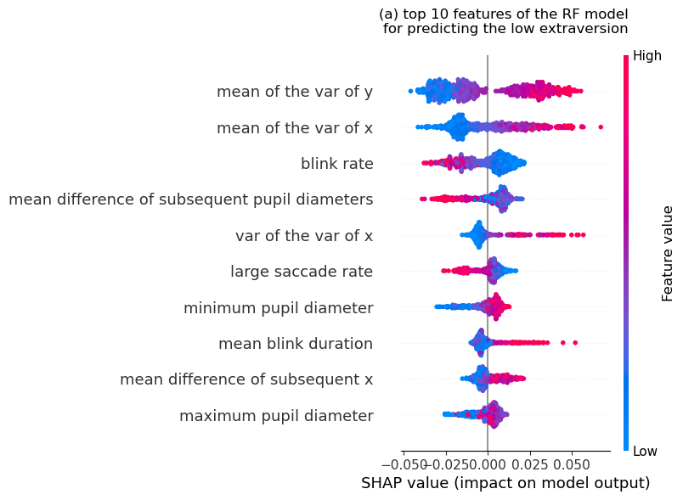


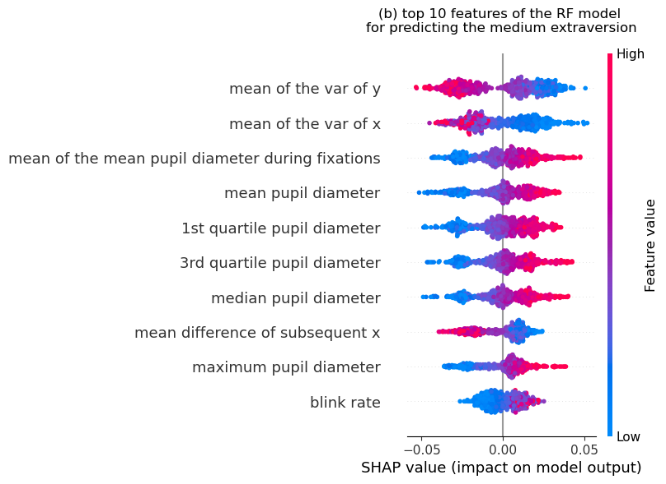


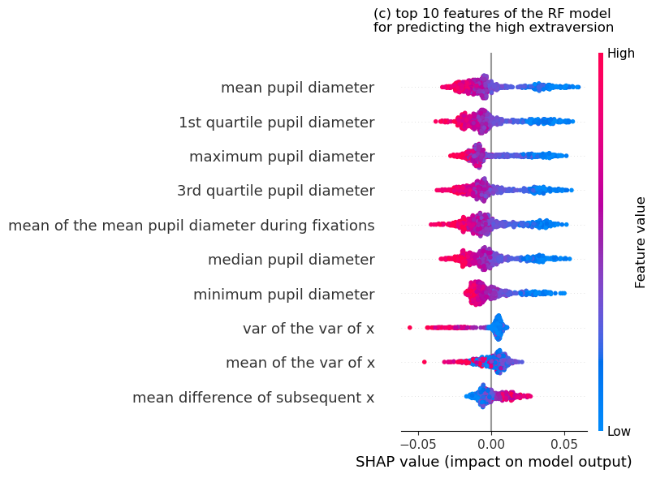

Supplement: Supplementary file 1 [file Data_Sheet_1.docx]
